# Dose coverage and breath-hold analysis of breast cancer patients treated with surface-guided radiotherapy

**DOI:** 10.1186/s13014-023-02261-0

**Published:** 2023-04-20

**Authors:** Janita Dekker, Marion Essers, Marcel Verheij, Martijn Kusters, Willy de Kruijf

**Affiliations:** 1 Instituut Verbeeten, Klinische fysica & instrumentatie, Postbus 90120, 5000 LA Tilburg, The Netherlands; 2 Department of Radiation Oncology, Radboud University Medical Center, Geert Grooteplein 32, 6525 GA Nijmegen, The Netherlands

**Keywords:** Surface-guided radiotherapy, Breath-hold analysis, Patient positioning, Breast cancer, Cone-beam computed tomography

## Abstract

**Background:**

Surface-guided radiotherapy (SGRT) is used to ensure a reproducible patient set-up and for intra-fraction motion monitoring. The arm position of breast cancer patients is important, since this is related to the position of the surrounding lymph nodes. The aim of the study was to investigate the set-up accuracy of the arm of patients positioned using SGRT. Moreover, the actual delivered dose was investigated and an extensive breath-hold analysis was performed.

**Methods:**

84 patients who received local or locoregional breast radiation therapy were positioned and monitored using SGRT. The accuracy of the arm position, represented by the clavicle position, was studied on the anterior–posterior kV-image. To investigate the effect of changes in anatomy and patient set-up, the actual delivered dose was calculated on cone-beam CT-scans (CBCT). A deformable registration of the CT to the CBCT was applied to deform the structures of the CT onto the CBCT. The minimum dose in percentage of the prescribed dose that was received by 98% of different CTV volumes (D98) was determined. An extensive breath-hold analysis was performed and definitions for relevant parameters were given.

**Results:**

The arm position of 77 out of 84 patients in total was successful, based on the clavicle rotation. The mean clavicle rotation was 0.4° (± 2.0°). For 89.8% of the patients who were irradiated on the whole-breast D98 was larger than 95% of the prescribed dose (D98 > 95%). D98 > 95% applied for 70.8% of the patients irradiated on the chest wall. Concerning the lymph node CTVs, D98 > 95% for at least 95% of the patients. The breath-hold analysis showed a mean residual setup error of − 0.015 (± 0.90), − 0.18 (± 0.82), − 0.58 (± 1.1) mm in vertical, lateral, and longitudinal direction, respectively. The reproducibility and stability of the breath-hold was good, with median 0.60 mm (95% confidence interval (CI) [0.66–0.71] mm) and 0.20 mm (95% CI 0.21–0.23] mm), respectively.

**Conclusions:**

Using SGRT we were able to position breast cancer patients successfully, with focus on the arm position. The actual delivered dose calculated on the CBCT was adequate and no relation between clavicle rotation and actual delivered dose was found. Moreover, breath-hold analysis showed a good reproducibility and stability of the breath-hold.

*Trial registration* CCMO register NL69214.028.19.

## Background

To accurately deliver radiotherapy, a reproducible patient set-up is important. Online imaging is commonly used to improve the set-up after the initial positioning [1]. However, posture differences can still occur [2–4]. During radiotherapy of the breast, patients are typically positioned with both arms lifted above the head. The degree of abduction of the arms is important, since this is related to the position of the surrounding lymph nodes [5, 6]. Bony landmarks, such as the thoracic vertebra (Th1-2) and the clavicle, are fixed to the lymph node regions. Cone beam CT (CBCT) can be used to check the set-up, however the field of view is often too short to visualize the treatment volume as well as the arms of the patient.

The conventional method of positioning breast cancer patients consists of tattoo points on the skin, which have to match to laser lines projected on the patient [7]. In spite of the use of a breast board with an armrest, it is difficult to exactly reproduce the arm position. A disadvantage of this laser-based set-up is that a patient can be correctly aligned based on the tattoo points, while the arms and other anatomic structures, like the breast itself, are slightly misaligned. Kapanen et al. [5] studied the arm position of patients treated by radiotherapy for breast cancer and in 65% of the patients an arm position correction was needed at least once during the course of treatment when using a wrist-hold fixation device.

Radiotherapy to the breast is often performed during breath-hold, which is a complicating factor in the set-up procedure. When patients are asked to hold their breath, the distance from the heart to the chest wall is maximized and the heart will receive less radiation [8–14]. Moreover, breath-hold is also beneficial for the lung tissue when the lymph nodes are treated [15].

An emerging modality in radiotherapy is the use of surface-guided radiotherapy (SGRT) for patient positioning and intra-fraction motion monitoring. The clinical use has rapidly increased and SGRT is used for various treatment sites [16]. SGRT can be used to improve the set-up of the patient, since posture differences are visualized and can be corrected before the online imaging. Studies described in literature have shown the added value of SGRT for positioning, used for various body sites like breast, thoracic, abdominal, pelvic, and intracranial [17–23]. Additionally, skin marks are not necessary when using SGRT, which makes the treatment more patient-friendly.

SGRT is not only used for positioning of the patient, it is also used for intra-fraction motion monitoring. The deviation of the isocenter is used to assess the breath-hold of a patient. Exceeding the threshold values means that the depth of inspiration is not correct. Set-up and breath-hold analysis is useful, but in the end the dosimetric effect of set-up inaccuracies and intra-fraction motion during inspiration is what counts.

In this study, set-up, breath-hold, and dosimetry data of a large group of breast cancer patients positioned and monitored with SGRT, were studied. The first aim of this study was to investigate the set-up accuracy of the arm of patients positioned using SGRT, by reporting the clavicle position. Second, the actual delivered dose to the breast clinical target volume (CTV), and chest wall CTV, as well as to the lymph node CTVs, was determined and the relation with the arm position was examined. The third aim of this study was to determine the residual set-up error, accuracy, and reproducibility of the breath-hold using SGRT.

## Methods

### Patient characteristics

91 patients who received local or locoregional breast radiation therapy with voluntary moderate deep inspiration breath-hold were included in this study from September 2020 to August 2021. Table 1 summarizes the patient characteristics. 6 patients were excluded from participation before the treatment started, due to several reasons (treatment did not meet the inclusion criteria (4 patients), technical failure of the SGRT system during the CT-scan (1 patient), and 1 patient withdrew her consent). One patient was excluded after the first treatment fraction, since she was not able to hold her breath long enough and it was decided to apply radiation during free breathing. Hence, in total data of 84 treated patients were used for the analysis. Informed consent was given by the participants and the study was approved by the medical ethics committee METC Brabant (CCMO register NL69214.028.19).

**Table 1:** Radiotherapy parameters and characteristics of the patients (n = 84)

	Number of patients (%)
Patients who completed the treatment	84 (100%)
**Fractionation scheme**	
15 × 2.67 Gy	62 (73.8%)
20 × 2.67 Gy (simultaneously integrated boost (SIB))	22 (26.2%)
**Radiotherapy**	
Whole-breast	60 (71.4%)
Chest wall	24 (28.6%)
**Treatment volume**	
Axillary lymph nodes level 1–2	22 (26.2%)
Axillary lymph nodes level 3–4	23 (27.4%)
Axillary lymph nodes level 1–4	22 (26.2%)

	With SIB	Without SIB
Local	12 (14.3%)	5 (6.0%)
Locoregional, without internal Mammary lymph nodes (IMN)	9 (10.7%)	46 (54.8%)
Locoregional, with IMN	1 (1.2%)	11 (13.1%)
**Age at start treatment (years)**		
Mean ± SD	55 ± 10 years	
Median (range)	55 years (35–77 years)	

### Treatment technique

Patients were treated on a Varian TrueBeam™, Clinac iX™, or Trilogy™ machine using 6 or 10 MV photon beams. The treatment plans were generated using the external beam planning module in ARIA 15.6 (Varian Medical Systems, Palo Alto, CA, USA) with the Acuros dose calculation algorithm [24, 25]. For most local breast cancer patients the treatment technique consisted of two tangential dynamic IMRT beams. For a few patients, a 180° VMAT beam with a weight of 10% was added. For local breast cancer patients receiving a simultaneously integrated boost, the technique consisted of two tangential IMRT beams, combined with two boost IMRT beams or one 180° VMAT beam. A hybrid technique consisting of two tangential open beams (80% of breast dose) and three 60˚ VMAT arcs (20% breast dose and 100% nodal dose) was used for the locoregional patients. We required a minimum value of 95% of the PTV volume that received at least 95% of the prescribed dose. We required a mean heart dose of maximum 3 Gy and aimed to ≤ 1 Gy for right breast irradiation and ≤ 2 Gy for left breast irradiation. When the dose to the heart exceeded 3.2 Gy for one of these plans, a plan with only VMAT arcs was applied to reduce the heart dose [9]. Delineation of the target volumes was performed according to the ESTRO guidelines [26]. The CTV to planning target volume (PTV) margin was 5 mm in all directions, for all CTV’s. Moreover, the CTV and PTV are cropped at 3 mm under the skin, and thus aligned at the skin side.

### Optical surface scanning system

The IDENTIFY™ system (Varian Medical System, Palo Alto, CA, USA) was applied for patient set-up and intra-fractional patient motion monitoring. For patient set-up, first a time-of-flight camera was used to generate the whole-body set-up. An infra-red light signal was send and the time needed to detect the reflected light was measured to determine the distance to the object [22]. This technique was used to make a 3D reconstruction of the entire body of a patient and was especially useful to position the arms of the patient. The whole-body reference image was acquired after finishing the CT-scan, while the couch was in the lowest position. Secondly, for precise positioning stereo vision based cameras were used. Two cameras captured the structured light pattern that was projected on the object. Hence, the location of every unique point was determined. Real-time surface images were compared to the reference image, obtained from the CT-scan. A rigid registration algorithm calculated the deviations of and around the isocenter to bring the two surfaces into alignment. The SGRT system used in this study consists of three sets of stereo vision cameras. A daily QA procedure was performed to verify the isocenter alignment. Submillimeter accuracy was achieved for the three translational degrees of freedom and for each rotation [22].

### Patient set-up using SGRT

All patients were positioned on a breast board with an inclination of 7.5°, with the arms lifted above the head in an arm support. Patient set-up was performed during breath-hold. Since a CT-scan takes about 45s to obtain, the procedure was split and the scan was acquired during 3 breath-holds. The patient body outline on the CT-scan was used to generate a surface image that served as reference image during set-up. The CT-scan was also used to calculate the absolute couch position at the treatment machine, where the patient is positioned to match the surface of the patient with the reference surface [4]. Since the position of the couch is known, a CT scan during breath-hold is enough to be able to reproduce the breath-hold level and a free-breathing scan is not needed.

During patient treatment, optical surface scanning was used for set-up, instead of the conventional method of using tattoo points on the skin and laser lines. The patient was positioned at the couch and a whole-body surface scan was used for the initial patient positioning, especially concerning the arms, to make the final alignment of the patient easier. Subsequently, SGRT cameras were used for precise positioning, while the patient held her breath. A threshold value of 5 mm for vertical, lateral and longitudinal direction, as well as for vector deviation was used, and a threshold value of 2° for pitch, roll or yaw rotation was used during set-up. The online match procedure was performed using orthogonal kV-images. The focus was on the implanted radio opaque fiducial clips, inserted in the breast during surgery before radiotherapy, followed by a check of the patient anatomy. Once a week, an extended CBCT scan was acquired.

After the online match procedure, a couch displacement was performed to correct for setup errors. To make sure the breath-hold level did not change during the couch displacement, an MV-image in the medio-lateral direction was acquired as additional check whether the breath-hold was within 5 mm of the reference value, using the position of the chest wall. Repositioning was performed when the residual error was more than 5 mm. After the online match procedure and couch displacement, a new surface scanning reference image, also during breath-hold, was captured for patient monitoring during the treatment fraction of that day.

A region of interest including the breast, sternum, and caudal part of the contralateral breast was drawn and was used both for set-up and intra-fractional motion monitoring by the surface scanner. Patients were instructed by a spoken recorded command to hold their breath. The breath-hold was monitored by means of the chest wall surface excursion. If the isocenter deviation was outside the threshold values of 5 mm translation or 2° of rotation around any orthogonal axis, the radiation was manually interrupted by the radiation technologist. The patient was coached by the technologists to change the depth of inspiration and subsequently the radiotherapy session was continued.

### Set-up accuracy of the arm

The accuracy of the arm positioning of the patient was assessed by determining the clavicle position. The anterior–posterior kV-image was used to match the clavicle to the CT-scan and the rotation error was determined. Success of the treatment was defined when in no more than five (for the 15 fraction scheme) or six (for the 20 fraction scheme) treatment fractions the difference in clavicle rotation was more than 3°, and in maximal two fractions it exceeded 5°. Based on a preliminary investigation in breast cancer patients treated without SGRT, the null hypothesis was that the probability of success equals 0.8. The study was designed to prove that in more than 80% of the patient treatments the set-up was successful. Statistical power was based on a one-sided exact binomial test using statistical package R (online R code compiler [27]).

In this study, the online acquired kV-images were compared to the digitally reconstructed radiographs of the reference CT-scan in an offline match procedure. The rotation around the dorso-ventral axis was used as outcome measure for the clavicle set-up.

### Dose calculation

To investigate the effect of changes in anatomy and changes in patient set-up, the actual delivered dose was calculated. Once a week, during fraction 2, 7, 12, and 17 if applicable, an extended CBCT was acquired, a CBCT-CT registration was performed, and the delivered dose was calculated on the extended CBCT scan. In this way we simulate an online match procedure based on an online CBCT match which we introduced in our institute after the trial. To ensure that the dose calculation on the CBCT reflects real values, Hounsfield units of the lungs and breast tissue on the CT and CBCT were compared for three patients. The differences between the mean values of each region were less than 20 HU, resulting in an accurate dose calculation using the CBCT.

A deformable registration of the CT to the CBCT was applied to deform the structures of the CT onto the CBCT, using the Image Registration module in the Varian treatment planning system. The registration was checked manually, and if necessary, changes were applied to the acquired contours. Second, the dose to the CBCT was calculated using Varian Eclipse treatment planning system. A database with relevant dose-volume values was calculated by means of in-house written scripts using the Python dicompyler-core library and subsequent analysis was performed using MATLAB R2015b (The MathWorks, Inc., Natick, USA). The minimum dose in percentage of the prescribed dose that was received by 98% of the CTV volume (D98) was determined for the breast, chest wall, axillary lymph nodes, and internal mammary lymph nodes (IMN). The D98 was determined for the fractions during which an extended CBCT was made. To make an estimation of the dose coverage of the whole treatment for each patient, the dose coverage was averaged over all CBCTs.

To investigate whether there is a correlation between the clavicle position and the dose coverage, the D98 for the different CTVs was plotted against the clavicle rotation.

### Breath-hold analysis

With SGRT, the position of the chest wall was monitored, and this was reported as the translations and rotations around the isocenter. The deviations in vertical, lateral, and longitudinal direction as function of the time were used to analyse the breath-hold. The outcome parameters used to assess the quality of the breath-hold (BH) were the residual set-up error (RE), the reproducibility (R), and the stability (S). The RE per breath-hold *i* is defined as the mean displacement from the start of the breath-hold (t = 1) till the end (t = T):

$$RE_{i} = \frac{1}{T}\mathop \sum \limits_{t = 1}^{T} BH_{i}$$

The RE per treatment fraction *f* is defined as the average RE_*i*_ in that fraction*:*

$$RE_{f} = \frac{1}{N}\mathop \sum \limits_{i = 1}^{N} RE_{i}$$

with *N* is the total number of breath-holds per treatment fraction.

Reproducibility of a treatment fraction was defined as the consistency of the depth of the breath-hold, given by the standard deviation of the mean vertical displacement of each breath-hold *i* during a treatment fraction:

$$R_{f} = \sqrt {\frac{{\mathop \sum \nolimits_{i = 1}^{N} \left( {RE_{i} - RE_{f} } \right)^{2} }}{N}}$$

with *N* is the total number of breath-holds per treatment fraction.

Stability of a single breath-hold was defined by the standard deviation of the breath-hold level, according to the definition used by Reitz et al. [28]:

$$S_{i} = \sqrt {\frac{{\mathop \sum \nolimits_{j = 1}^{T} \left( {BH_{j} - RE_{i} } \right)^{2} }}{T}}$$

Stability of the breath-hold of a treatment fraction was defined by the mean of the breath-hold levels in a treatment fraction, given by:

$$S_{f} = \frac{1}{N}\mathop \sum \limits_{i = 1}^{N} S_{i}$$

with *N* is the total number of breath-holds per treatment fraction, BH_j_ is the breath-hold level at timestamp *j,* and *T* is the number of measurements of a breath-hold.

**Fig. 1:**
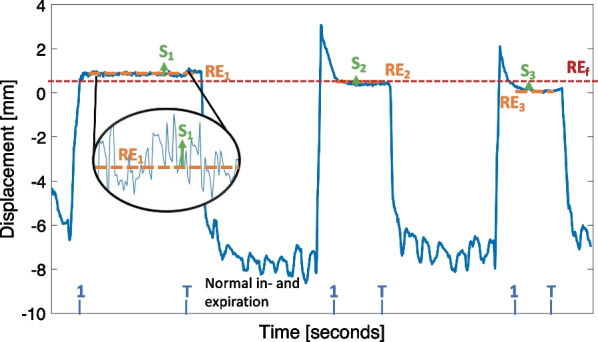
Plot of a breath-hold signal, visualizing the breath-hold parameters RE_i_, RE_f_, and S_i_

The breath-hold parameters RE_i_, RE_f_, and S_i_ are visually explained in Fig. 1.

## Results

### Set-up accuracy of the arm

**Fig. 2:**
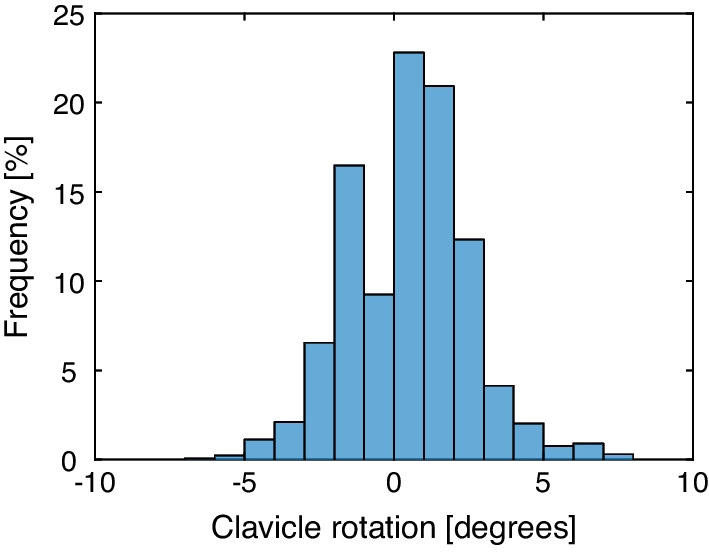
Histogram of clavicle rotation around the dorso-ventral axis determined on the kV-image of all fractions of all patients. A correction for the left and right arm was applied, hence a positive value of the clavicle rotation refers to an adduction of the arm, while an abduction is represented by a negative value

In 77 out of 84 patients the set-up of the arms was successful, based on the clavicle position determined on the kV-image. Figure 2 shows the histogram of the clavicle rotation of all fractions. A positive value of the clavicle rotation means an adduction of the arm (i.e. the arm was positioned too low), while a negative value means an abduction of the arm with respect to the planning CT. The mean clavicle rotation was 0.4° (95% confidence interval (CI) [0.3–0.5]), with a standard deviation of 2.0°. This means that some patients experience difficulties with positioning their arm high enough in the positioning aid. According to the study design, we performed a one-sided exact binomial test. With a probability of success of 91%, we can reject the null hypothesis that the probability of success equals 0.8 (p value is 0.003, 95% CI for the probability of success is [0.85–1.0]), meaning that the arm positioning using SGRT was accurate.

### Dose calculation

**Fig. 3:**
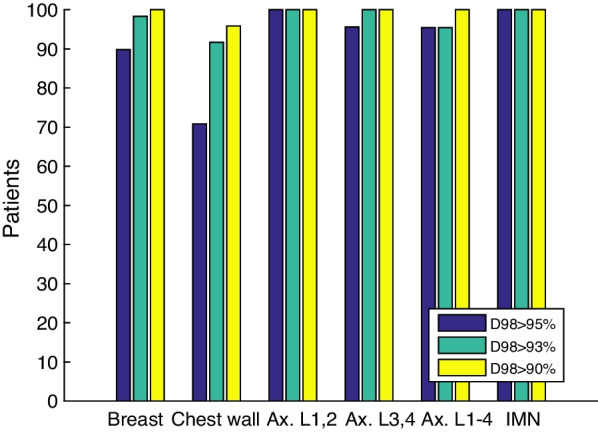
Bar graph showing D98 for a threshold of 95%, 93%, and 90% of the prescribed dose, for the breast CTV (60 patients), chest wall CTV (24 patients), axillary lymph node level 1–2 CTV (22 patients), axillary lymph node level 3–4 CTV (23 patients), axillary lymph node level 1–4 CTV (22 patients), and IMN (12 patients) CTV

The bar graph in Fig. 3 shows the percentage of the patients where the minimum dose to 98% of the volume was more than 95% (D98 > 95%), 93% (D98 > 93%), or 90% (D98 > 90%) of the prescribed dose for the six CTV volumes. For example, regarding the axillary lymph nodes level 3–4, the percentage of the patients where at least 98% of the CTV received at least 95% of the prescribed dose, was 95.7%, while for 100% of the patients D98 was at least 90% of the prescribed dose. Concerning the threshold D98 > 95% of the prescribed dose, the lowest value was for the chest wall, with 70.8% of the patients.

From the 60 patients in total who were irradiated on the breast, for 6 patients D98 ≤ 95%. Two of these patients received a dose between 92 and 93% on 98% of the CTV. For the other four patients the D98 was between 94 and 95%. For one of the patients irradiated on the chest wall out of 24 in total, the D98 was with 89.9% just smaller than the threshold of 90%, while for another patient D98 was 90.2%. For both patients, this was caused by a difference in patient posture and by patient deformations, resulting in a displacement of the CTV outside the high-dose area. Five patients irradiated on the chest wall had a D98 between 93.7% and 94.9%. For all patients irradiated on the axillary lymph nodes and on the IMN D98 > 95%. Regarding the axillary lymph nodes level 1–4 one patient out of 22 in total had a D98 ≤ 95%. The D98 for fraction 12 of this patient was 87.9%, due to difficulties performing breath-hold during set-up.

### D98 versus clavicle rotation

**Fig. 4:**
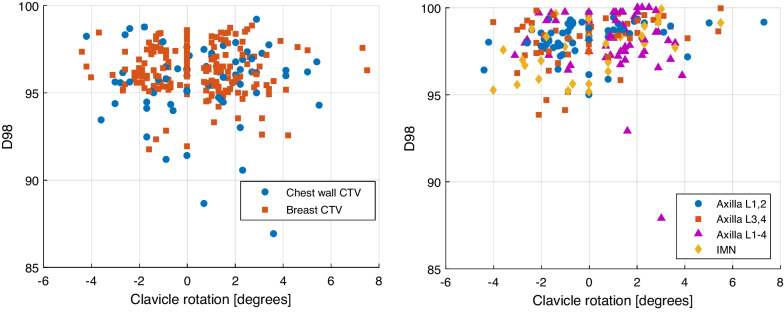
D98 plotted against clavicle rotation for the breast and chest wall CTV (left figure) and for axillary lymph node level 1–2, axillary lymph node level 3–4, axillary lymph node level 1–4, and IMN CTV, per treatment fraction. A correction for the left and right arm was applied, hence a positive value of the clavicle rotation refers to an arm position that was too low, while a high arm position is represented by a negative value

The effect of the clavicle rotation on the dose coverage of the different treatment volumes is shown in Fig. 4. From these plots no clear relation can be found between the dose coverage and a clavicle rotation. In Fig. 4 the D98 is plotted per treatment fraction, instead of D98 per patient treatment as in Fig. 3 and was for most fractions more than 95% of the prescribed dose. The lower values for the dose coverage were caused by a difference in patient posture and/or by patient deformations, resulting in a displacement of the CTV outside the high-dose area.

### Breath-hold analysis

In total 5859 treatment beams delivered in 1213 treatment fractions were analysed.

**Fig. 5:**
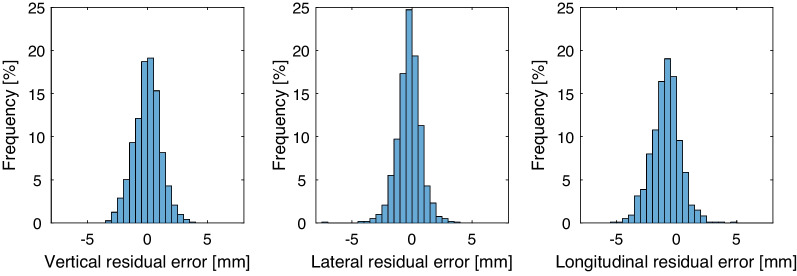
Histograms of the RE_f_ in vertical (left), lateral (middle) and longitudinal (right)

The residual error per treatment fraction *f *(RE_f_) as determined by the average of the mean vertical, lateral, and longitudinal deviation of each breath-hold is shown in the bar graph in Fig. 5. The mean ± standard deviation was − 0.015 ± 0.90 mm, − 0.18 ± 0.82 mm, − 0.58 ± 1.1 mm in vertical, lateral, and longitudinal direction, respectively. A negative value of the longitudinal translation means that the patient is shifted in cranial direction, which can be compensated by a caudal shift of the couch. In 95%, 96%, and 88% of the treatment fractions the absolute RE_f_ was < 2 mm in vertical, lateral, and longitudinal direction, respectively. Since the threshold value on the vertical displacement of the isocenter was set to 5 mm, larger deviations were not observed.

**Fig. 6:**
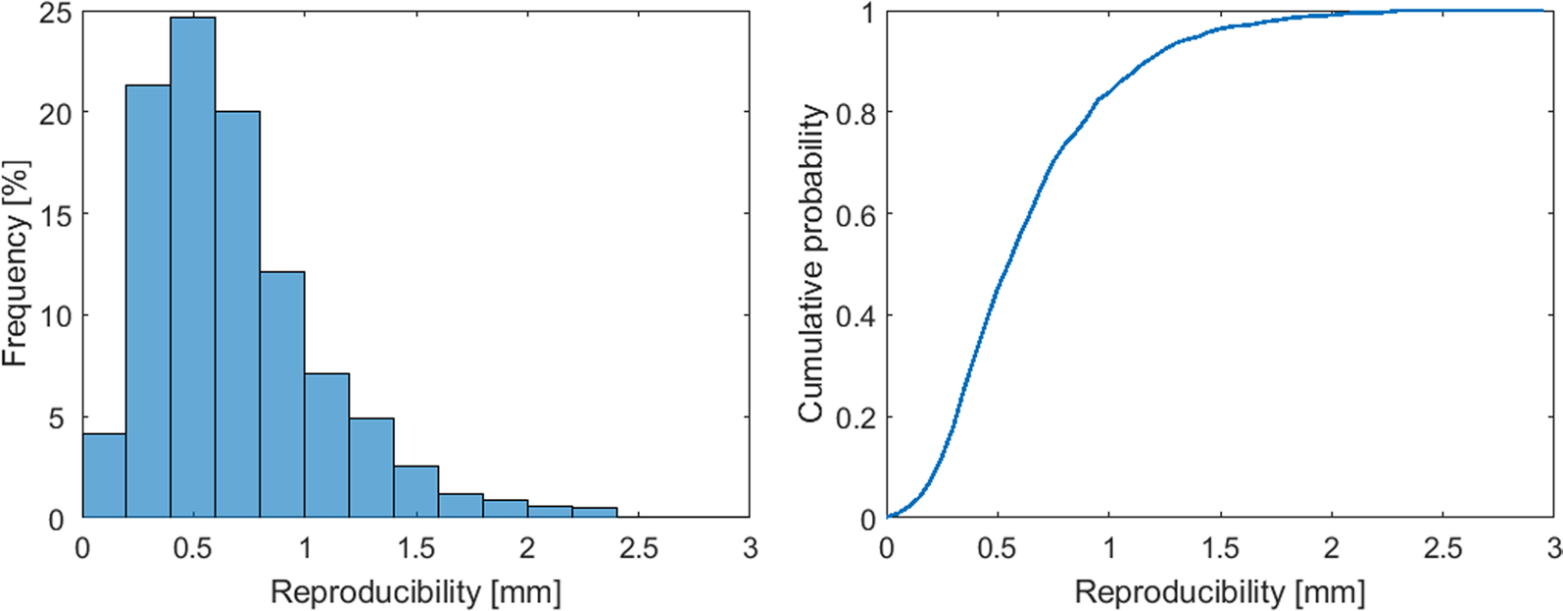
Histogram and cumulative probability distribution of the R_f_

Figure 6 shows the reproducibility per treatment fraction R_f_ and the cumulative probability distribution of the R_f_ less or equal to a certain value in vertical direction. The reproducibility values were averaged over all patients and showed a median value of 0.60 mm (95% CI [0.66–0.71] mm).

**Fig. 7:**
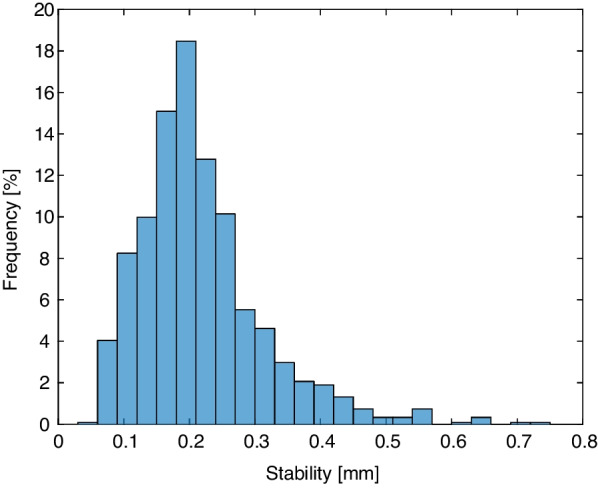
Histogram of the S_f_

The stability per treatment fraction S_f_ is shown in the histogram in Fig. 7. The stability values were averaged over all patients and showed a median value of 0.20 mm (95% CI [0.21–0.23] mm).

## Discussion and conclusions

This study focused on the effect of SGRT on the set-up accuracy, especially of the arm and the breast itself, and its effect on the actual delivered dose to the different treatment volumes. SGRT was applied for precise positioning, for intra-fraction motion monitoring, and for breath-hold analysis. Studies in literature already proved that a good correlation exists between set-up errors determined with CBCT compared to SGRT for breast cancer patients [29, 30]. Since online imaging is performed, only differences in patient posture and patient deformations can cause differences between the planned and delivered dose distribution. With SGRT, these posture differences can be minimized. The advantage over surface-guided based set-up is that the whole surface of the patient is used for positioning instead of only a few reference points, while the set-up accuracy remains the same [31–33] or even improves, since more information (like the arm position), can be used for patient set-up [34]. Kügele et al. studied the set-up deviations of tangential and locoregional breast cancer patients who were positioned using either tattoo points or SGRT. The set-up deviations significantly decreased when using SGRT [35]. In this study, the accuracy of the arm position was analysed for a large group of patients, as opposed to the patient set-up analysis reported in studies described in literature. The study was designed to prove that 80% of the patients can be positioned successfully and with 91% it can be concluded that the positioning was successful (*p* = 0.003).

The dose coverage of different treatment volumes was calculated on the CBCT, by performing a deformable registration of the CT to the CBCT. Note that this resulted in an under- or overestimation of the total dose, since only three or four CBCTs were used for each patient and these serve as estimation for the entire treatment. However, the weekly made CBCTs were spread over the treatment, hence any swelling or difference in patient anatomy that arose during the treatment was included. Moreover, the total number of patients in the study should be sufficient such that the CBCTs are representative for the whole treatment. According to the margin recipe of Van Herk, 90% of the patients should receive 95% of the prescribed dose to the CTV [36]. In accordance with Dutch national standards, a coverage of 98% of the CTV is required. Applying this to our results, we conclude that the applied CTV to PTV margin was adequate for the lymph node and IMN CTVs, since at least 95.5% of these patients received a dose of at least 95% to 98% of the volume. Therefore, these CTV to PTV margins might even be decreased. The degree of decreasing this margin is subject of further research.

In this study the coverage of the chest wall was slightly lower compared to the other treatment volumes. This can be explained by the shape of the CTV. Compared to the breast CTV, the chest wall CTV is elongated and thin and has a small volume. As a result of patient posture and patient deformations, the part of the CTV that falls outside the high dose area is relatively large, compared to a larger volume. A lower coverage of the breast and chest wall CTV compared to the lymph nodes CTVs can be explained by the breast and chest wall CTV being more deformable than the lymph node region. It shows that differences between the planned and actual patient position have a larger effect on the dose coverage in those regions than on the coverage of the lymph nodes. Therefore, we suggest focusing on the breast and chest wall CTV in the online match procedure rather than on the lymph node regions. There was one patient with a D98 of the axillary lymph nodes level 1–4 of 96.7%, 92.9% and 87.9% for fraction 2, 7, and 12, respectively. The corresponding clavicle rotation was 0.8°, − 1.6°, and − 3°. This is the only patient out of 22 who were irradiated on the axillary lymph nodes level 1–4, where the dose coverage of the axillary lymph node level 1–4 decreased when the clavicle rotation increased. For the other treatment volumes, such a correlation was not found.

The residual set-up error represents the degree the breath-hold level matches with the reference. This was determined per treatment fraction by averaging over all breath-holds and the results showed a mean residual setup error of − 0.015 (± 0.90) mm, − 0.18 (± 0.82) mm, − 0.58 (± 1.1) mm in vertical, lateral, and longitudinal direction, respectively. Figure 5 shows that the displacement in longitudinal direction was larger compared to the vertical and lateral direction and the distribution was not symmetrical. This implies that during the treatment fraction the patient surface was shifted too far in cranial direction. We speculate that this might be due to a lift of the back of the patient during breath-hold. In a study of Gnerucci et al. [37] the mean treatment shifts for locoregional treatments, with an SGRT threshold of 5 mm, were − 0.49 mm, 0.10 mm, and − 0.83 mm in vertical, lateral, and longitudinal direction, respectively. Comparable to our results, a longitudinal shift of the patient in cranial direction was observed. Gnerucci et al. concluded that an increased amount of air was inhaled during the breath-hold. Our results showed that in at least 88% of the treatment fractions the absolute residual error was < 2 mm. Penninkhof et al. determined the residual error per treatment beam and reported similar results: in their study in at least 85% of the treatment beams the residual error was < 2 mm [38].

Some studies in literature defined reproducibility as the maximum difference between mean inspiration levels per beam within a treatment fraction [10, 28, 38–40]. Hence, outliers have a large effect on the reproducibility. Therefore, we decided to define reproducibility by the standard deviation of the mean inspiration level. The difference in definition can be an explanation of the higher value compared to our results. In the study of Xiao et al. reproducibility was calculated as the median value of the 5th–95th percentile range of the displacement during a breath-hold [41]. Still their reproducibility of 2.2 mm was more than we report.

The residual set-up error, reproducibility, and stability values depend on the motion monitoring threshold that is applied, as a lower threshold value reduces the maximum shift and improves the stability [42]. Our results showed a breath-hold reproducibility and stability of 0.60 mm (95%-confidence interval (CI) [0.66–0.71] mm) and 0.20 mm (95% CI 0.21–0.23] mm), respectively, and this is better than what was reported by other studies in literature, where the reproducibility ranged from 0.5 to 2.3 mm [10, 28, 39–41]. Stability values of 0.3 mm and 1.5 mm were reported [10, 28, 39, 41]. Cerviño et al. applied a motion monitoring threshold of ± 1.5 mm and their results showed a reproducibility of 0.5 mm and a stability of 0.7 mm in patients receiving visual feedback using video goggles [10]. However, the number of breast cancer patients included in the study was low with 5 patients. Therefore, the results might not be applicable to a larger group of patients, since a small group of patients might not be representative. Kügele et al. also applied a motion monitoring threshold of ± 1.5 mm and their results showed a reproducibility of 1 mm [40]. A slightly higher threshold value of mean ± 2 mm, which was individually selected per patient, was applied by Reitz et al. [28]. This resulted for 103 patients in a mean reproducibility of 1.3 mm (95% CI [0.5–2.6] mm). The stability of the breath-hold, defined as standard deviation of the breath-hold level was 0.3 mm (95% CI [0.1–0.9] mm). An even larger value of the threshold was used by Xiao et al. with values up to 3.5 mm [41]. They reported a worse reproducibility up to 2.2 mm in a study including 58 patients. The reported stability was smaller than 0.7 mm. In the study of Hamming et al. no threshold was applied at all and this resulted in a reproducibility of 2.3 mm and a stability of 1.5 mm in 18 patients [39]. In our study threshold values of ± 5.0 mm and ± 2.0° were applied. According to the good reproducibility and stability, it is observed that the threshold values could be reduced. Based on the results of our study, the threshold value was already decreased in our institute to 3 mm and 2°, but an even lower value can be considered.

Two measures of stability were compared in a study of Reitz et al.: the standard deviation of the breath-hold level and the linear deviation based on the linear fit model [28]. It was concluded that there was no difference. Since the parameters can be seen as comparable, we decided to use the simpler standard deviation as measure for stability. Our results showed a stability of 0.22 mm. This is comparable to the value of 0.3 mm that was reported by Reitz et al. [28]. Hamming et al. reported a stability of 1.5 mm [39]. The stability was calculated by the difference between the start and end position of each breath-hold. Hence, a deviating value at the end of a breath-hold has a larger effect compared to calculating the standard deviation. This may explain the differences with our results.

As a result of the method using the absolute couch position in our institute, the set-up deviations without SGRT are difficult to compare with other departments and the set-up deviations are already small. To be able to make a comparison between patients treated without and patients treated with SGRT an unrealistically large number of patients would be needed to achieve enough statistical power. Therefore, a limitation of this study is that the results are based on one particular patient group that was positioned and monitored using SGRT and that no comparison was made with a patient group not treated with SGRT. Nevertheless, we conclude from the results that the patients included in the study were treated adequately, since the reproducibility and stability of the breath-hold of the patients in this study was at least comparable to studies described in literature. Moreover, the dose coverage of the treatment volumes meets the requirements.

To conclude, the use of SGRT results in small set-up deviations of the clavicle of breast cancer patients. SGRT has turned out to be a valuable addition to the method of patient positioning in our institute using absolute couch coordinates, where the couch position needed for the set-up is calculated from the CT. The actual delivered dose calculated on the CBCT was adequate. No relation between clavicle rotation and actual delivered dose was found. Moreover, analysis of the breath-hold showed a good reproducibility and stability of the breath-hold.

## Data Availability

The datasets generated and analysed during the current study are not publicly available due to the privacy of the subjects and are stored by Institute Verbeeten. The data is available from the corresponding author on reasonable request.

## Abbreviations

CBCT: Cone-beam computed tomography
CI: Confidence interval
CTV: Clinical target volume
IMN: Internal mammary nodes
D98: Minimal dose to 98% of volume
PTV: Planning target volume
R: Reproducibility
RE: Residual set-up error
S: Stability
SGRT: Surface-guided radiotherapy
